# Designing a Clinician-Centered Wearable Data Dashboard (CarePortal): Participatory Design Study

**DOI:** 10.2196/46866

**Published:** 2023-12-05

**Authors:** Shehjar Sadhu, Dhaval Solanki, Leslie A Brick, Nicole R Nugent, Kunal Mankodiya

**Affiliations:** 1 University of Rhode Island Kingston, RI United States; 2 Brown University Providence, RI United States

**Keywords:** digital health, wearables, smart watch, smartwatch, symptom monitoring, mobile health, mHealth, participatory design, stress management, monitoring, eHealth, wearable technology, remote monitoring, physical stress, psychological stress, stress, data interpretation, visualization, questionnaire, decision-making, mobile phone

## Abstract

**Background:**

The recent growth of eHealth is unprecedented, especially after the COVID-19 pandemic. Within eHealth, wearable technology is increasingly being adopted because it can offer the remote monitoring of chronic and acute conditions in daily life environments. Wearable technology may be used to monitor and track key indicators of physical and psychological stress in daily life settings, providing helpful information for clinicians. One of the key challenges is to present extensive wearable data to clinicians in an easily interpretable manner to make informed decisions.

**Objective:**

The purpose of this research was to design a wearable data dashboard, named CarePortal, to present analytic visualizations of wearable data that are meaningful to clinicians. The study was divided into 2 main research objectives: to understand the needs of clinicians regarding wearable data interpretation and visualization and to develop a system architecture for a web application to visualize wearable data and related analytics.

**Methods:**

We used a wearable data set collected from 116 adolescent participants who experienced trauma. For 2 weeks, participants wore a Microsoft Band that logged physiological sensor data such as heart rate (HR). A total of 834 days of HR data were collected. To design the CarePortal dashboard, we used a participatory design approach that interacted directly with clinicians (stakeholders) with backgrounds in clinical psychology and neuropsychology. A total of 8 clinicians were recruited from the Rhode Island Hospital and the University of Massachusetts Memorial Health. The study involved 5 stages of participatory workshops and began with an understanding of the needs of clinicians. A User Experience Questionnaire was used at the end of the study to quantitatively evaluate user experience. Physiological metrics such as daily and hourly maximum, minimum, average, and SD of HR and HR variability, along with HR-based activity levels, were identified. This study investigated various data visualization graphing methods for wearable data, including radar charts, stacked bar plots, scatter plots combined with line plots, simple bar plots, and box plots.

**Results:**

We created a CarePortal dashboard after understanding the clinicians’ needs. Results from our workshops indicate that overall clinicians preferred aggregate information such as daily HR instead of continuous HR and want to see trends in wearable sensor data over a period (eg, days). In the User Experience Questionnaire, a score of 1.4 was received, which indicated that CarePortal was exciting to use (question 5), and a similar score was received, indicating that CarePortal was the leading edge (question 8). On average, clinicians reported that CarePortal was supportive and can be useful in making informed decisions.

**Conclusions:**

We concluded that the CarePortal dashboard integrated with wearable sensor data visualization techniques would be an acceptable tool for clinicians to use in the future.

## Introduction

### Overview

According to surveys conducted by the American Hospital Association and Center for Disease Control, the use of eHealth systems has seen a dramatic increase from 2010 to 2020 [1,2]. eHealth offers the integration of modern daily used technologies such as wearable devices and smartphones for the diagnosis and longitudinal symptom monitoring of chronic conditions such as Parkinson disease, dementia, diabetes, hypertension, cardiovascular diseases, and mental disorders [3-6]. Wearable technologies such as smart watches, smart rings, and smart garments are becoming popular because they enable the gathering of quantified health information in daily life environments such as homes, nursing homes, and assisted living facilities [6]. The health data from wearables include but are not limited to physiological parameters (heart rate [HR], blood oxygen saturation, respiration rate, and body temperature) and activity and behavioral data (step count, calories burned, active periods, and sedentary periods) [3-6].

One of the key challenges is to make these massive multimodal data (collected from wearables) interpretable to clinicians for clinical decision support. Owing to busy schedules and time constraints, clinicians seek meaningful and interpretable methods for wearable data visualizations. To ensure usability, it is important to integrate clinicians’ needs and preferences in the process of designing interpretable dashboards. A clinician-centered dashboard demands the development of new ways to analyze and visualize data for clinicians who are looking for specific health outcomes relevant to their patients and practices. Therefore, it is essential to understand the workflow of clinicians. For example, clinicians use specific software systems (electronic health records [EHRs] and patient portals) to provide information and make decisions. It is important not to burden clinicians with excessive data visualization. At the same time, the dashboards must not omit important features derived from the continuous stream of wearable data.

To address these challenges and meet the needs of clinicians, our research was aimed at designing a data visualization dashboard, CarePortal, to display wearable device data to clinicians. CarePortal is envisioned as a potential solution that can reduce the complexity of wearable sensor data interpretation and visualization and help clinicians make informed decisions more efficiently. This study aimed to answer the following broad research question: “What are the vital elements of a data analytics dashboard that can help to visualize, analyze, and interpret patient wearable data for clinicians to support an informed decision-making process in an easy interpretable way?”

Contributions of this research are as follows:

- *Participatory design with clinicians*: we aimed to design and test a user interface (UI) for the CarePortal data dashboard that visualizes symptomatic health data from a smartwatch in an interpretable manner. We used a participatory design approach involving interactions with clinicians (with backgrounds in clinical psychology and neuropsychology). A 5-stage participatory design workshop study was conducted that allowed the evolution of the CarePortal dashboard through the iterative feedback of clinicians about the ways they prefer to visualize wearable device data. The workshops started with an understanding of the needs and workflow of the clinicians. Subsequently, the dashboard design was iterated from low-fidelity prototypes to an interactive web application.
- *A web application dashboard architecture of longitudinal symptomatic data of wearable smartwatch*: we used a wearable data set collected from 116 adolescent participants who were exposed to trauma [7]. Participants wore a Microsoft Smart Band 2 that logged physiological sensor data such as HR. A total of 834 days of HR data were collected. In this study, we developed a data processing pipeline for wearable device data (from Microsoft Band) and the CarePortal dashboard software architecture.

### Background and Related Works

Participatory design is a methodology that promotes the iterative engagement of end users in the design process of solutions [8-10]. In participatory design, each phase is planned by reflecting on the results of the previous phase with respect to the participants’ contributions. In general, participatory design can be divided into several phases, including understanding the needs and problems of end users, brainstorming ideas for possible solutions, developing prototypes, testing and refining prototypes, and evaluation. One of the key benefits of participatory design is that end users have a say in the design process and are often included as co-designers because they are invited to proactively collaborate and brainstorm potential solutions [11].

In the areas of eHealth and digital health, participatory design processes are widely used to ensure that the challenges of end users are addressed in end products that are designed and tested interactively for high user acceptance and satisfaction. Seals et al [12] inquired about the need for clinicians to visualize wearable data on remote gait assessments in people with multiple sclerosis. This was a participatory design study involving researchers as participants from different domains, such as human-computer interaction, biomedicine, neurology, and rehabilitation. Their participatory design process resulted in insights pointing toward the need for quantitative sensor data that can help track specific rehabilitation goals. The study also reported the need to balance between a quick overview and a detailed understanding of the critical information. This study demonstrates how participatory design can help identify key insights into what matters most to end users and form recommendations on potential solutions.

Regarding patient-centered design, participatory design processes are widely adopted to meet the needs of patients by targeting customized health apps for various applications, such as care planning [13], self-management of asthma [14], and procedure education [15]. However, the use of participatory design is limited in meeting the needs of clinicians who are an integral part of health care and demand to understand the status quo of their patients. Clinicians are busy professionals who see and treat many patients daily. They follow standard protocols and workflow. They need to make clinical decisions based on the information gathered from patients and self-reported evaluations (often retrospective reports, daily logs, or notes). Modern technologies such as eHealth and wearable devices can add significant burden to clinicians if these technologies are not integrated within their workflow according to the clinician’s needs.

This study offers insights into how wearable device data can be integrated into their workflow. This study aims to investigate the visualization of wearable device data gathered daily, as clinicians do not have the time and energy to see the raw data because of their busy schedules. Accordingly, it is important for software and app developers to assess clinician preferences for the visualization of data, ensuring that the end product will facilitate informed decisions. This study leveraged participatory design processes with clinicians to develop a finalized web application dashboard, which is a product of iterative feedback and testing from clinicians as end users.

## Methods

### Overview

This participatory design study involved the creation of a software framework for the development of rapid webapp iterations. Hence, our research was divided into 2 parts. First, we instrumented a preliminary software architecture for CarePortal that can host and process the wearable device data. The software architecture also allowed us to design and iterate web application interfaces to visualize real-world wearable data. Later, the interfaces were used during participatory design workshops with clinicians.

### Dashboard Software Architecture

#### Wearable Sensor Data Set

**Figure 1:**
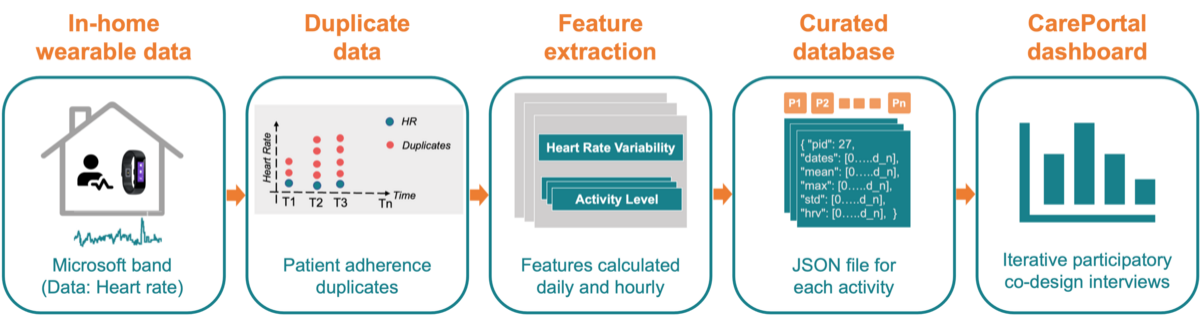
A visual data processing pipeline followed to create the CarePortal data dashboard for clinicians. JSON: JavaScript Object Notation.

The CarePortal dashboard was designed to visualize smartwatch sensor data previously collected in a clinical study (in the years 2017-2020) [7]. This study included 116 patients who presented to the emergency department following traumatic injury. The Microsoft Smart Band 2, paired with a custom-made companion Android app, was deployed in the participant’s daily life for 2 weeks. The app was developed using the Microsoft Software Development Kit to collect sensor logs. The app collected data from the photoplethysmography sensor, which was then stored in comma-separated values files. A total of 834 days of HR data were collected. A data preparation and processing pipeline (discussed in detail in the following sections) was developed to meet the dashboard requirements raised by clinicians. Figure 1 presents an overview of the data processing and dashboard deployment pipelines.

#### Wearable Data Set Preparation

This section describes the data preparation methods applied to the wearable sensor data set. This includes identifying and removing duplicate data from the data set.

#### Duplicate Data: Identification and Removal

To check for duplicate data in this data set, the timestamps for each day for each participant were analyzed. We found that 5 (out of 116 adolescents) participants had over 50% of duplicate data for unknown reasons. However, as this data set was longitudinal and each participant performed the study for several days, not all the day’s data were duplicated. We found that at least 1 day of data was duplicated for these 5 participants. Therefore, CarePortal dropped the days for which ≥50% of the data were found, which was 9 days in total out of the total 834 days of data. After removing the duplicates, the data were forwarded to the processing pipeline.

#### Feature Extraction: Wearable Sensor Data Processing

After assessing the requirements of clinicians in workshops 1, 2, and 3 (discussed in later sections), we incorporated HR-based features into our processing pipeline. To process and extract features from the wearable sensor data set, we focused on (1) HR-based statistical features, (2) HR variability (HRV)–based features, and (3) HR-based activity levels.

#### HR-Based Statistical Features

On the basis of inputs from clinicians, we selected the maximum, minimum, SD, and average HR as statistical features. These features present simple statistical metrics that can easily be interpreted by clinicians. We calculated the HR value’s daily and hourly maximum, minimum, SD, and average. Daily statistics will allow clinicians to quickly analyze several days of data, and hourly statistics will allow clinicians to see the details of specific events of interest.

#### HRV-Based Features

We used this metric in the CarePortal dashboard, as research suggests that HRV is a relevant metric to measure stress [16,17]. Daily and hourly HRV were calculated. In addition, we computed the maximum, minimum, and average HRV for the day [18]. Equation 1 shows the calculation of HRV. Similar to the HR, the daily and hourly HRV were calculated.



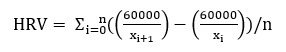



where *n* represents the total number of samples, *i* indicates the increment counter variable, and x_i_ represents the HR value at the ith index.

#### HR-Based Activity Levels

**Figure 2:**
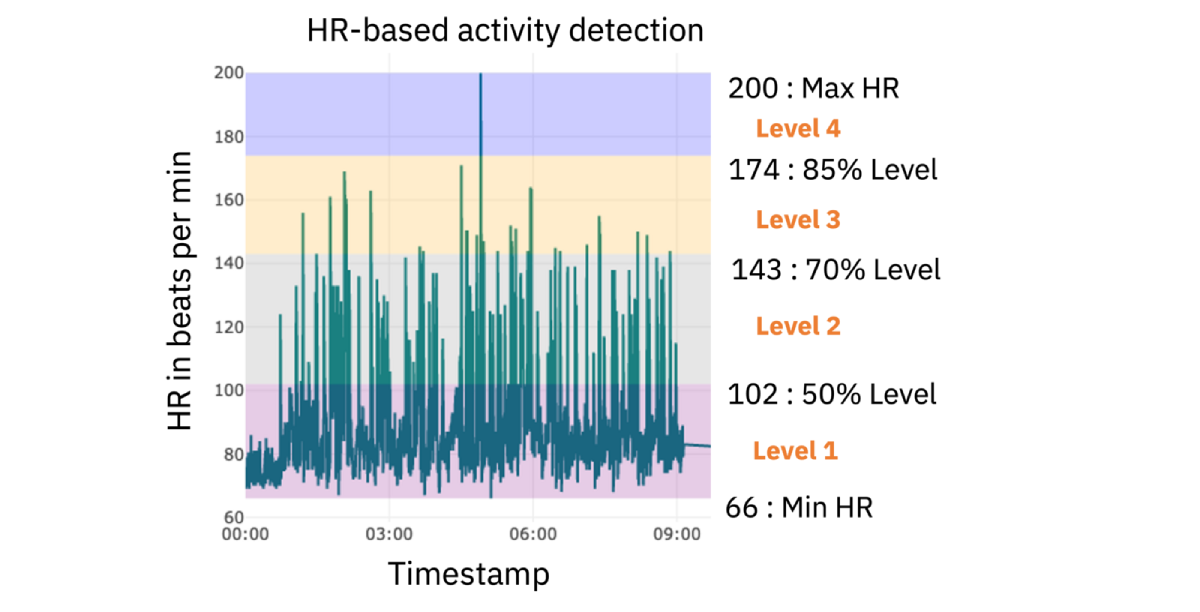
Raw heart rate (HR) data showing septate activity levels. Four activity levels are shown: level 1, ≤66 beats per minute (bpm; pink highlight); level 2 indicating 50% (gray highlight); level 3 indicating 70% (yellow highlight); level 4 indicating 85% (purple highlight).

For clinicians, it was important to know when the HR was high and when it was low, which can be attributed to the high or low activity or stress levels. This was observed in the phase 1 interviews. To achieve this, we calculated HR-based activity levels and followed the guidelines of the American Heart Association [19]. The maximum HR (max HR) was computed using the formula given in equation 2. To compute HR_MAX_, age was selected as 15 (average age of adolescents). On the basis of HR_MAX_, we calculated 4 different activity levels. These levels were as follows: ≤50% activity level, 50% to 70% activity level, 70% to 85% activity level, and ≥85% activity level. Figure 2 shows the different activity classes for the sample HR data based on different activity levels. Figure 2 presents the raw heart data collected from a single day’s activity for 1 participant.

Max HR = 220 – Age **(2)**

Level 1: HR≤(max HR) × 50% **(3)**

Level 2: (max HR) × 50%<HR<(max HR) × 70% **(4)**

Level 3: (max HR) × 70%<HR<(max HR) × 85% **(5)**

Level 4: HR≥(max HR) × 85% **(6)**

#### CarePortal Dashboard Development

The dashboard web application development process involved an interdisciplinary team of UI or user experience (UX) designers and software engineers. The dashboard design was divided into three parts:

1. *Data visualization (frontend)*: the CarePortal dashboard was developed using the Python Flask micro web framework. The Flask framework combined HTML, CSS, JS, and Python. This dashboard used the JavaScript version of the Plotly framework for visualizations. This version of Plotly allowed us to customize the built-in features that were not required by clinicians.
2. *Curated database (backend)*: the CarePortal dashboard used Google Drive to store the curated database and application programming interface from the Google Cloud Platform to query the data from Google Drive. The database was formatted in a JavaScript Object Notation file format, where each metric was separated by a key value pair. In this JavaScript Object Notation format, each day and hours data values were separated.
3. *Dashboard deployment*: we used Apache 2 Web Server Gateway Interface configurations on a Linux server to deploy the dashboard application. Once the application was deployed, a custom domain name was used. This domain name was then sent to clinicians who tested the dashboard web application.

### CarePortal: Participatory Design Study

To design the CarePortal dashboard web application, it was of utmost importance to consider feedback from end users (clinicians) during the design process. Participatory design allowed us to create the interfaces with them in an iterative process that helped us understand which metrics (discussed above) were useful for clinicians to analyze patient data.

**Figure 3:**
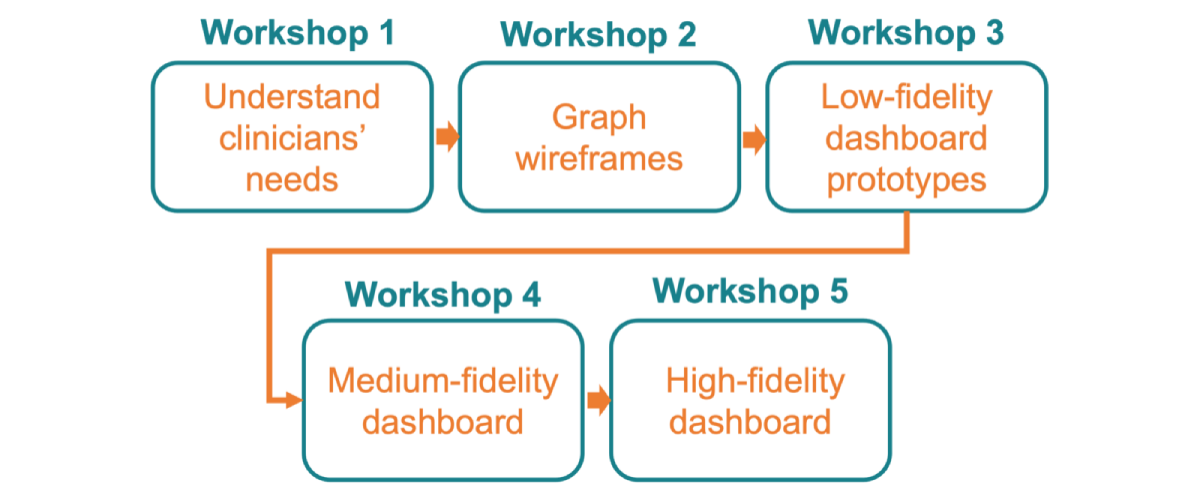
Five workshop stages of participatory design. These workshops were conducted serially throughout the study. In the first 3 workshops, 3 clinicians were recruited. In workshops 4 and 5 (deployment workshops), 6 clinicians were recruited.

Five one-on-one design workshops with clinicians were conducted in this study. Workshops 1, 2, and 3 were conducted sequentially, whereas workshops 4 and 5 were conducted after a few months of analyzing and reflecting on the data from the first 3 interviews and developing the web dashboard accordingly. This participatory design process is shown in Figure 3. Owing to the restrictions of the COVID-19 pandemic, all workshops were conducted virtually using the Zoom platform. Each workshop lasted for approximately 35 to 40 minutes. To analyze the qualitative data from this workshop, the research team carefully took notes and coded clinicians’ responses from the Zoom recordings.

### Ethical Considerations

This study was approved by the institutional review board (IRB) of the University of Rhode Island (#1776865-1). Video recordings of clinicians were kept on a local hard drive and only researchers approved by the IRB had access to these videos. As this research was exempt from IRB approval, we obtained a digital consent signed by participants for this study. The participants volunteered to participate in this study, and no compensation was provided.

### Clinician Recruitment

Clinicians with a background in psychology and neuropsychology with clinical practice in behavior therapy were recruited for this study. Clinicians in these areas of expertise were selected to best match the presenting concerns of the research population receiving behavior therapies. Clinicians from hospitals and clinics in and around Rhode Island, the United States, were recruited via an email campaign. This email included a short abstract about the study and the time commitment required by the clinicians.

### Workshop 1: Understanding Clinician Needs

The main aim of workshop 1 was to gain an empathetic understanding of clinicians’ daily practice and the tools they use during their treatment process.

For this, questions regarding their behavior therapy practice, treatment understanding, and use of technology in day-to-day clinical practice were asked. Textbox 1 presents a list of questions asked during this process. This was a conversational workshop. The workshop began by discussing the meeting agenda, followed by a permission request to record the meeting. To analyze the qualitative data from this interview, methods for creating user personas were applied [20]. For each clinician, a user persona was created that included a short biography, pain points (user concerns), goals, and the treatment process. Textbox 1 presents a list of questions asked during this workshop.

**The list of questions for workshop 1 (n=3).**

**Part 1: behavioral health understanding**

1. How would you describe your practice?
2. How does your therapy work?
3. What type of patient populations do you serve?
4. How often do you see your clients?
5. Do you interact with your clients outside of office hours? if so, how?

**Part 2: treatment understanding**

1. Can you walk me through the initial evaluation process?

**Part 3:** **technology use in clinical practice**

1. Do you see wearable technology playing a role in your therapy now or in the future?
2. How did the COVID pandemic impact your practice? In what ways did it change?

### Workshop 2: Graph Wireframes

**Figure 4:**
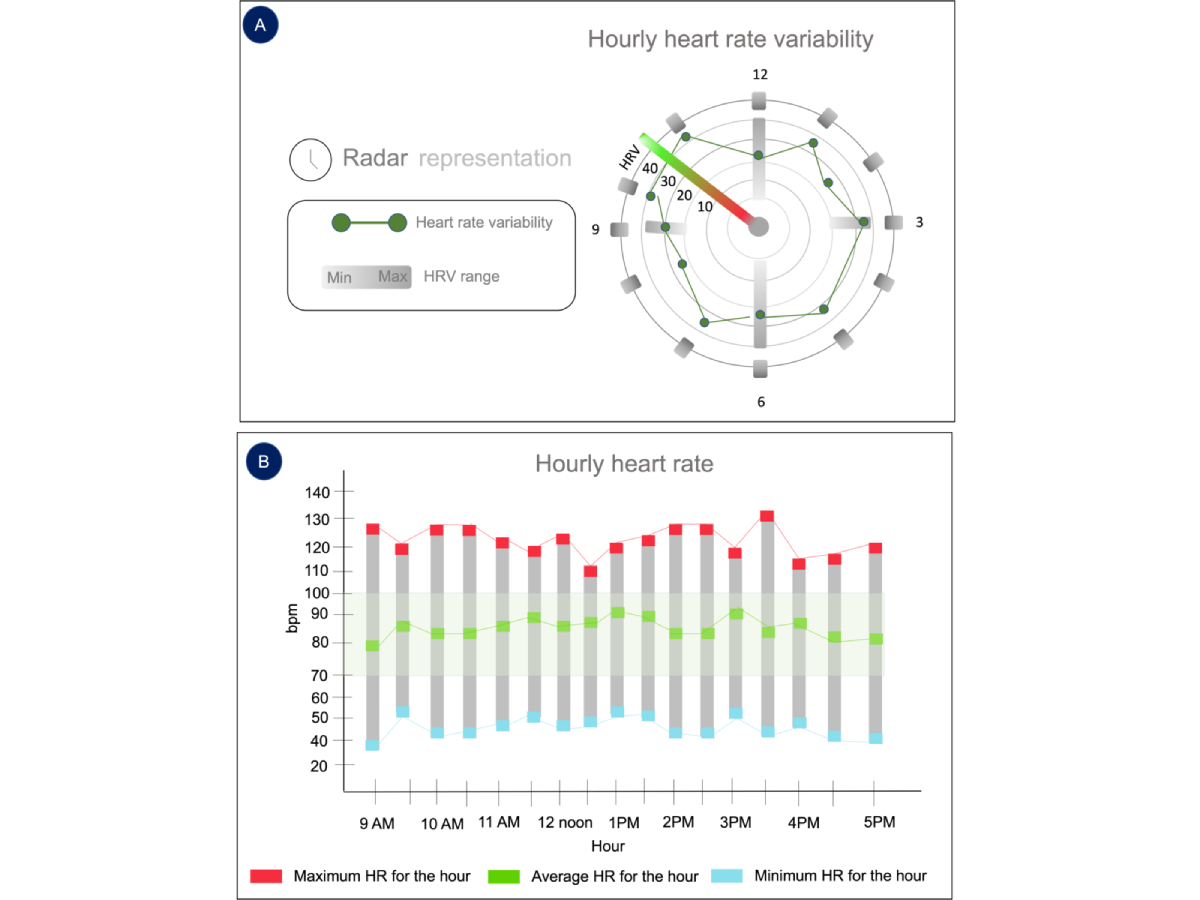
Workshop 2 graph wireframes: (A) presents a radar plot for hourly heart rate (HR) variability parameter and (B) a bar graph for the same.

The second workshop incorporated initial low-fidelity wireframes. This workshop was aimed at understanding how clinicians would like to visualize patients’ health data collected from wearables. The workshop protocol consisted of 2 parts. The first part was an interactive conversation in which we asked questions to understand what kind of health data are helpful for clinicians. In the second part, the aim was to understand what types of graphs or visualizations were preferred by clinicians. For this purpose, low-fidelity wireframes were designed using the Figma prototyping tool (developed by Figma Inc). These wireframes were presented using slides via Zoom screen sharing. The wireframes (shown in Figure 4) consisted of different types of graphs, such as (1) a bar plot representing the hourly HR and (2) a radar plot representing the hourly HR variability.

### Workshop 3: Low-Fidelity Dashboard Prototypes

**Figure 5:**
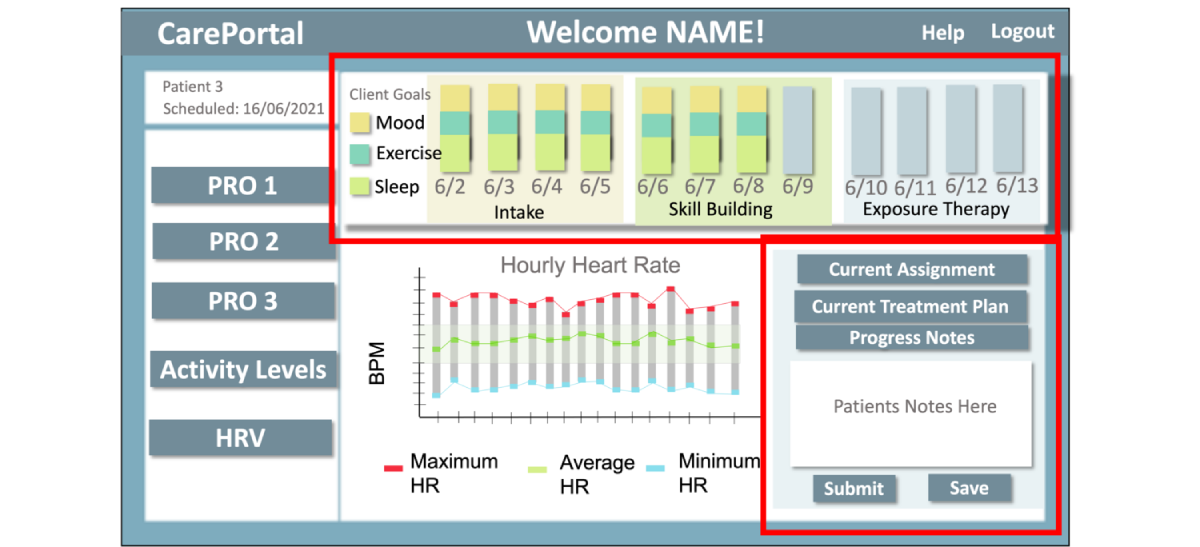
Workshop 3: low-fidelity prototype. HR: heart rate; HRV: heart rate variability; PRO: patient-reported outcome.

The main aim of this workshop was to show clinicians low-fidelity prototypes of the CarePortal dashboard and to better understand the requirements of clinicians from those prototypes. For this purpose, 3 low-fidelity prototypes of CarePortal were presented to the clinicians. These prototypes were created after analyzing the requirements of the clinicians in previous workshops. Figma (developed by Figma Inc) was used for prototyping and allowed us to quickly iterate the design prototypes. This was presented to clinicians via Google Slides using the share screen feature of Zoom. Figure 5 shows this prototype version.

### Workshop 4: Medium-Fidelity Interactive Prototype

In this workshop, the aim was to showcase the CarePortal dashboard prototype to clinicians (so they can use it remotely) and receive feedback on the prototype. A link to the high-fidelity dashboard prototype, a functional Python Flask web app, was provided to the clinicians. In this workshop, clinicians could interact directly with the dashboard using their own machines. This prototype was developed based on the feedback received from previous workshops, and the suggested UX features (eg, hovering over legends for graphs and loading spinners) were implemented on the dashboard. Subsequently, we describe the specific features and navigation of the CarePortal dashboard in detail. To understand clinicians’ perspectives, during this workshop, clinicians shared their screens so that the researcher could observe their interactions and clinicians could provide feedback while moving their mouse pointer over the screen. A detailed feature description of this prototype version is provided in the *Dashboard UI* section.

### Workshop 5: High-Fidelity Interactive Prototype

In this workshop, the primary aim was to have clinicians interact with different versions of graphs to give us feedback on their experience and preference.

This version of the CarePortal dashboard was designed based on feedback from clinicians on the deployment of workshop 4. Three data metrics were presented to the clinicians: (1) HR, (2) HRV, and (3) activity levels (HR based). Options for different types of graphs were provided for each data type, which were implemented in the form of a carousel on the dashboard. At the end of this interview, the User Experience Questionnaire (UEQ) was administered to clinicians to quantitatively analyze the usability of the dashboard [21]. On providing permission to record the session, participants were asked to share their screen. This allowed researchers to observe how clinicians navigate this dashboard. The workshop began with a guided tour of the CarePortal dashboard. Once the workshop was completed, a link to a Google Form consisting of the UEQ questions was provided. Similar to workshop 4, we also implemented UX features in this dashboard version, including hover-over legends and loading spinners. The following sections describe the changes made to the CarePortal dashboard UI based on feedback.

### Dashboard UI

#### Overview

**Figure 6:**
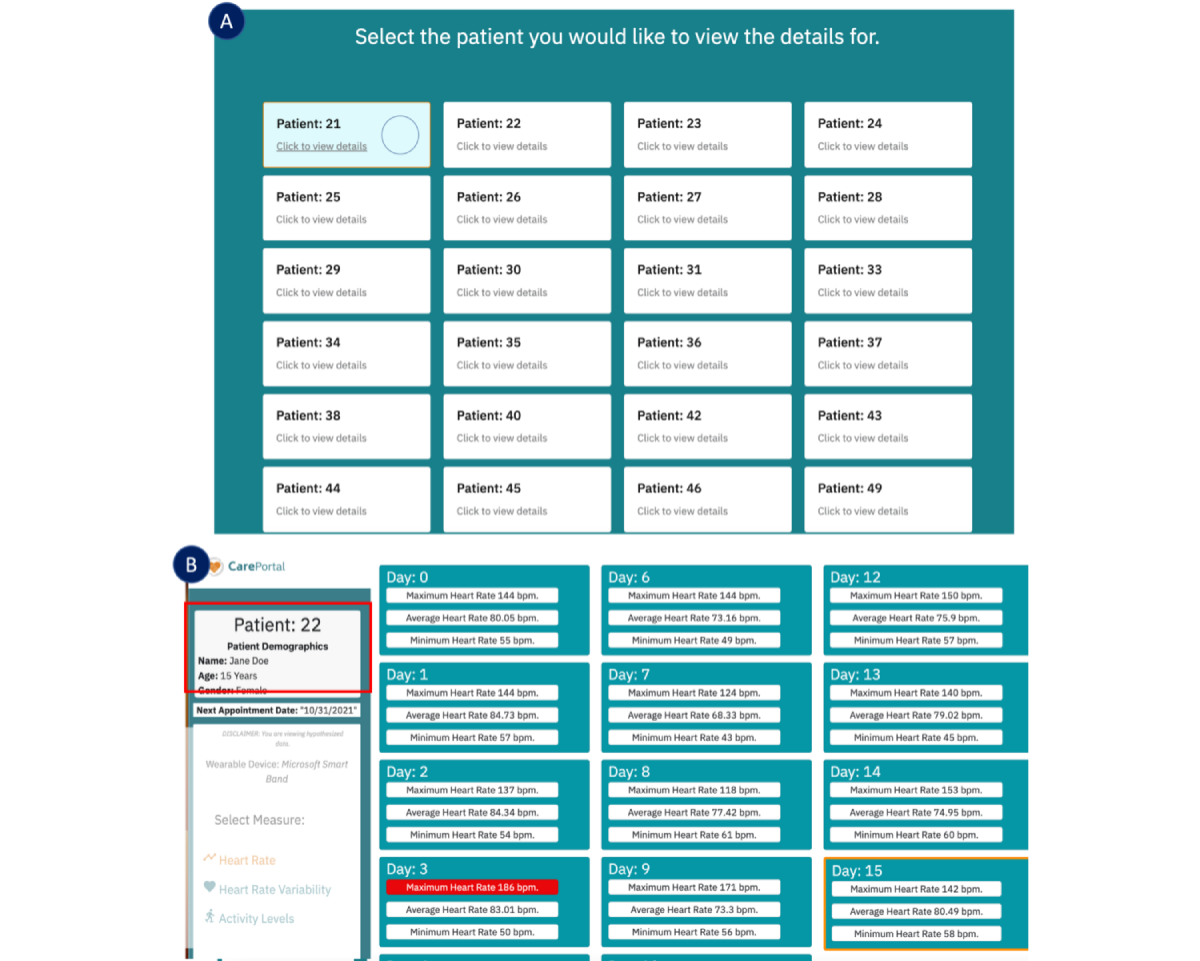
Deployment prototype 1 overall progress page displaying daily statistics of heart rate data: (A) patient selection page and (B) overall progress page.

**Figure 7:**
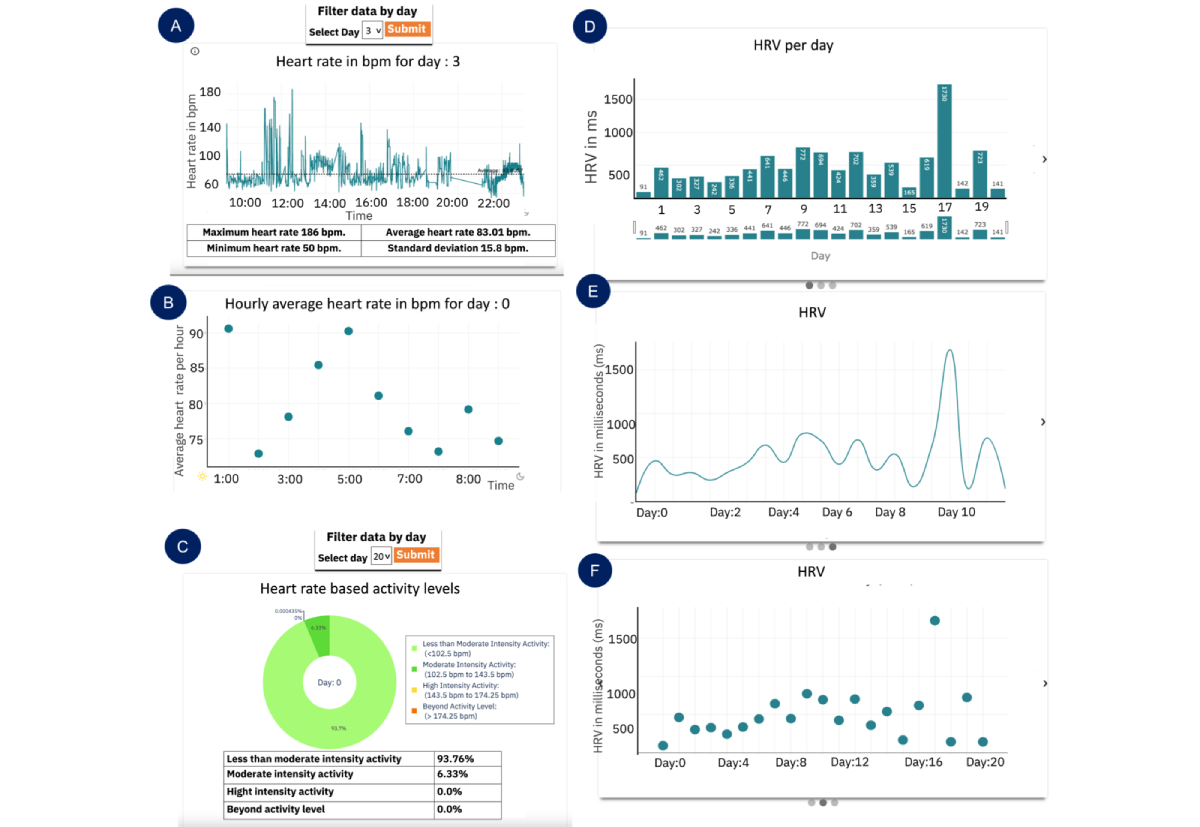
Panels A, B, and C represent data from workshop 4: deployment (n=6); panels D, E, and F represent data from workshop 5: deployment 2 (n=5). Heart rate page: (A) raw heart rate–based activity levels; (B) hourly heart rate statistics; (C) a pie chart representing activity levels; (D) a bar plot for heart rate variability (HRV); (E) a line plot for daily; and (F) a scatter plot for daily HRV. bpm: beats per minute.

**Figure 8:**
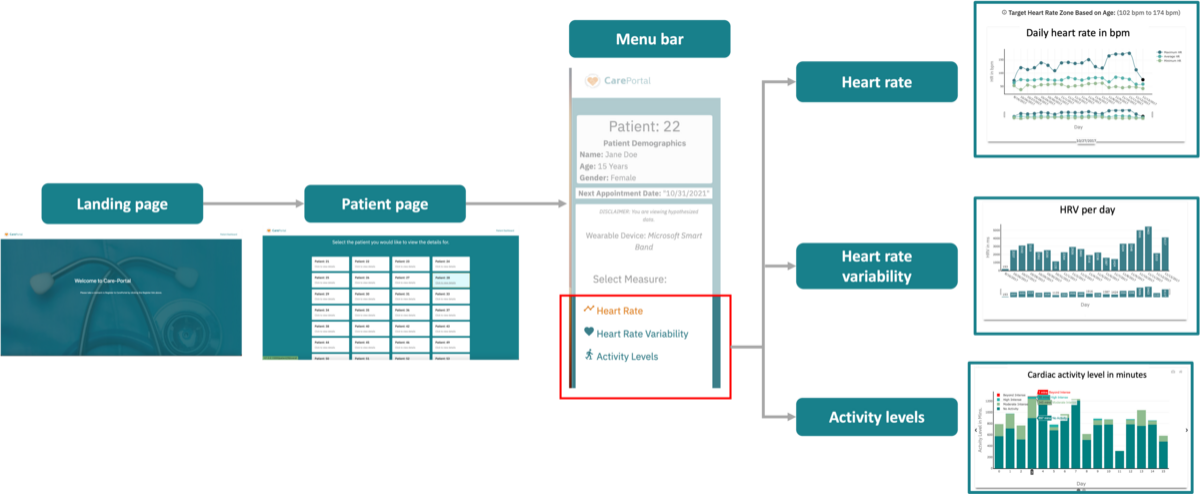
The navigational chart of the finalized CarePortal web application.

In this section, we describe the UI of the deployed CarePortal dashboard. This prototype encompassed three sections: (1) the overall progress page, (2) the HR page, and (3) the activity level page. Within each page, a carousel was implemented so that clinicians could view different types of graphs on the 3 pages for the same information. Figures 6 and 7 show the prototypes of the app. Figure 8 presents a navigational chart of the finalized CarePortal web application.

#### Overall Progress Page

Once the clinician selected a patient on the patient’s page, they were directed to the overall progress page. The overall progress page displayed all patient data, including the maximum, minimum, and average HRs for all days, in a card view. If the maximum HR of a particular day was above level 3 (>174 beats/min, ie, 85% max HR), which was calculated based on the details discussed in the *Wearable Sensor Data Processing* section, the maximum HR bar within that card view was coded as red. A detailed overview of the overall progress page is shown in Figure 6.

#### HR Page

On the HR page, the idea was to provide an overview of the HR data by presenting the following: (1) the raw data plot of HR over the whole day and (2) a scatter plot of hourly averages to present information in aggregate form. A day filter option was provided to clinicians to filter data for each day. Figure 7A displays the raw HR plot, and Figure 7B presents the hourly HR plot.

#### HR Activity Levels Page

On the activity level page, a pie chart (shown in Figure 7C) was presented, showing the percentage activity levels calculated based on HR. Four activity levels were derived, that is, (1) less than moderately intense (level 1), (2) moderately intense (level 2), (3) high intensity (level 3), and (4) beyond intense activity (level 4). Similar to the HR page, a filter option was provided to clinicians to filter the daily data. The percentage levels were additionally presented in a table, as it was difficult to see small percentages in the pie chart.

#### Loading Spinner

Feedback was given to users once they clicked on the “click here to view details” link while the page was loaded in the form of a loading spinner. This is shown in Figure 6A in a green circle (Patient 21).

#### On Hover Color Change

When the mouse hovered over a particular card view, the border turned orange to indicate the card that was selected. This was implemented in both patients’ selection (Figure 6A) and overall progress page (Figure 6B).

#### Navigation Bar (Navbar)

The hover method was implemented in the navbar of the web application, which made the navbar link turn orange when clicked on it. This was implemented in both the horizontal and vertical navbars. When the user logged in, they were greeted by their username on the vertical navbar in the top-left corner, as shown in Figure 6B.

## Results

In this section, we discuss the results of the participatory design workshops with clinicians along with qualitative and quantitative analysis.

### Clinician Recruitment

Recruitment resulted in a total of 8 clinicians from Rhode Island Hospital and University of Massachusetts Memorial Health. As summarized in Table 1, a total of 3 clinicians were recruited in workshops 1, 2, and 3, whereas 6 clinicians were recruited in workshops 4 and 5. One clinician dropped out in workshop 3. A similar case occurred in workshop 5, where 1 participant withdrew owing to personal emergencies. Therefore, we did not include their comments in this section. Clinicians recruited in workshops 1, 2, and 3 were different from those recruited in workshops 4 and 5; however, Participant 1 was the same for all workshops.

**Table 1:** Participant demographics (clinical background and demographics of each clinician in this study).

Participant number	Gender	Clinical background	Specialty	Workshop 1	Workshop 2	Workshop 3	Workshop 4	Workshop 5
P1	Female	Clinical psychology	Anxiety disorders	Yes	Yes	Yes	Yes	Yes
P2	Male	Neuropsychology	Neurodegenerative disorders	Yes	Yes	No	—^a^	—
P3	Female	Music therapy	Mental health disorders	Yes	Yes	Yes	—	—
P4	Male	Neuropsychology	Cognitive impairments	—	—	—	Yes	Yes
P5	Male	Neuropsychology	Epilepsy	—	—	—	Yes	No
P6	Male	Clinical psychology	Family therapy	—	—	—	Yes	Yes
P7	Female	Clinical psychology	Emergency department	—	—	—	Yes	Yes
P8	Female	Clinical psychology	Adolescents with stress	—	—	—	Yes	Yes

^a^Did not participate in the corresponding workshop.

### Workshop 1: Understanding Clinician Needs (Insights)

In this workshop, we gained insights into the treatment process for anxiety disorders. We found that the first step in the treatment process was to collect the patient’s prior history, symptoms, and active problems. This information was entered into an EHR system. Participant 1 uses *Simply Practice* as the EHR system. The technology used daily by Participant 2 includes phone and fax, whereas Participant 3 uses Spotify and HIPAA (Health Insurance Portability and Accountability Act)–compliant Google Drive. Participant 1 followed a 16-week protocol on average. Clients are scheduled to see a clinician on a weekly basis and each meeting lasts for approximately 45 minutes to 1 hour. During each session, the clinician took notes following the Data Assessment and Plan notes. Data Assessment and Plan is a form of clinical note–taking technique. In the first 8 weeks of therapy, a skill-building process is followed. In this process, a clinician teaches patients how anxiety feels in the body and what to do if an anxiety event occurs. Participant 1 mentioned, “We try to identify what their anxious patterns are, what their anxious thoughts are, and then replacing those with more positive coping like thoughts.” In the next 8 weeks, exposure therapy with response prevention was administered. In this way, a fear hierarchy is created, which is a list of things the client is concerned about. Participant 1 described to us what that means explicitly:

We make a list of all the things that make the client anxious, so let’s just say for example, they are in because they are really afraid of their best friend’s dog, and wouldn’t go over to the best friend’s house, and this was causing a lot of problems because the families were friends. So, what we do is create a fear hierarchy and identify things that make the client scared and how we are going to work on those in the session. We would look at pictures of calm cute dogs and on a scale of 1-10 the fear is maybe a 2 out of 10. We would work on this until the client told us their anxiety went down from a 2 to a 1.

One of the pain points during this process for Participant 1 was the ability to track how anxiety felt when the client was doing their therapy homework. As Participant 1 said, “We ask the clients to do homework. It would be interesting to be able to take that piece of it and look at what their anxiety feels while they are doing their homework, because all we have now is self-reports from the client, and it is probably not that accurate.”

Participant 2 uses prior information about patients’ health as their primary source of data to diagnose them. Therefore, Participant 2 does not see patients on a regular basis; instead they prepare a treatment plan and recommendations for patients after diagnoses. Participant 3 with a background in music-based therapy mentioned the use of software such as Spotify and HIPAA–compliant G-suit applications, for example, Excel for data analysis and Google Docs for note taking. Participant 3 also mentioned that one of the limitations of using Excel for data analysis is that they have a larger amount of data to analyze, and it is not possible to look at everything via Excel sheets. Participant 2 mentioned, “Currently we use Excel to store and conduct our data analysis however an app would be more helpful to track quantifiable data.” This motivated us to create data visualization mechanisms that can be used to easily interpret data and provide actionable insights to clinicians. We describe the clinician’s biography, pain points, general goals, and daily used technology in Table 2.

**Table 2:** Workshop 1 analysis.

Participant no.	Brief biography	Pain points	Goals	Preferred health parameters
P1	P1 is a Doctor of Philosophy in clinical psychology who works for a clinical psychology clinic and sees children. Specializes in seeing children with anxiety disorder.	Ability to track how anxiety felt while patients did their homework. Metrics other than self-reports are important as children are reporting these and there is room for error.	Help her clients get better through evidence-based therapy who are diagnosed with anxiety disorder.	Heart rate Breathing rate
P2	P2 is a neuropsychologist who sees children ranging from preschool to high school age. Conducts neurological evaluations such as ability in attention, memory function, motor coordination, judgment, reasoning, and emotional and mental health concerns.	Does not have any prior health information before conducting evaluation. Wants to corelate sleep with self-reports for last 3 mo.	Diagnose patients and create a treatment plan for them.	Last 3 mo of sleep data Cardiovascular exercises with target zones
P3	P3 is a music therapist who sees children with a wide variety of mental health issues. Patients’ therapy goals are related to nonmusic goals. Music is the means to get to those goals. She works with a variety of persons such as patients, caregivers, and family members who help to increase awareness of brain conditions and reduce social stigma.	HIPAA^a^-compliant EHR^b^ system for music therapy. A system that can store music playlists. Excel sheets are not ideal, an app interface can be more helpful in analyzing quantifiable health information.	Help clients with mental health issues using music. Interested in the use of rhythm and gait.	Freezing of gait Stress scales

^a^HIPAA: Health Insurance Portability and Accountability Act.

^b^EHR: electronic health record.

### Workshop 2: Graph Wireframes—Insights

In this workshop, 2 types of graph wireframes were shown to clinicians, as shown in Figure 4. All 3 clinicians preferred the bar plot version of the graph, and the radar chart was described as hard to interpret. Participant 1 said, “It will be important to keep the visuals very simple. Because these are master’s level (degree type) clinicians, they have not necessarily got a lot of research training, they also don’t have a lot of time, so this has to be something they can look at very quickly and extract meaningful information.” Participant 1 also suggested that instead of displaying only the maximum, minimum, and average numbers, “it would be helpful if you presented the number of minutes there was a maximum.” Participant 3 mentions that “instead of just showing maximum, minimum and average a baseline to compare the patient data with will be helpful.”

A daily view of the data was preferred by both Participant 1 and Participant 2 in this version. Participant 1 explicitly said, “instead of hourly heart rate, a daily/weekly view would be better.” For the radar chart, Participant 1 said, “Looks cool, but most clinicians are trained on a horizontal axis. This would probably confuse people.” In the second prototype design, we incorporated a bar plot and a daily view of the HR data.

### Workshop 3: Low-Fidelity Dashboard Prototypes (Insights)

In this workshop, clinicians aimed to understand how mood, exercise, and sleep were tracked. Participant 2 asked, “Can you tell me more about what is on the left mood, exercise, and sleep? What is the input?” They wanted to know the sources of their data. Clinicians also wanted to obtain a daily view of the data. Participant 2 asked, “For this one, what are number of spikes always per week?” In addition, Participant 1 shared similar thoughts and said “I think clinicians are going to use it across the day because there would be multiple instances across the day.” This led us to create 2 views for the deployment study, the first being daily data presentation and the second being the hourly data presentation in the high-fidelity prototypes.

Overall, in our first 3 workshops, we learned significantly about the needs of clinicians and how they would like wearable data to be visualized. Here, we summarize the key considerations for the deployment prototypes in the next phase of interviews: (1) a daily view of the data is preferred by clinicians and (2) simpler visualizations are preferred by clinicians, for example, the bar plot version is easier to interpret than the radar plot.

### Workshop 4: Medium-Fidelity Interactive Prototype (Insights)

In the following sections, we describe the insights gained from workshop 4. We divide this section by presenting an analysis of the overall progress page, the HR page, and the activity levels page.

#### Insight 4.1: Overall Progress Page (“A Graphical Presentation Is More Interpretable”)

In this view, clinicians shared the unanimous concern that numbers were too much to interpret; Analyzing data in this view was difficult. Participant 7 critiqued saying the following:

Numbers are important to have, they probably would not be the first thing I would put forward because they are hard to digest. First thing I noticed was there was a lot of data on the page, so it took me a second to get oriented.

Graphs showing trends were more desirable for this view. Participant 4 explicitly described to us how the graph might look in the overall progress page: “It would be interesting to present a view like the graph view of the heart rate tab. Maybe in the overall progress tab you could have a graph with x axis as the days and three lines for max, min, average HR. That could be kind of cool to see that.” This suggestion was incorporated into the second high-fidelity prototype. A detailed overview of the overall progress page is presented in Figure 6B.

#### Insight 4.2: Overall Progress Page (“Clickable Card View Navigation”)

On the overall progress page, the card’s view seemed clickable. Participant 4 said, “If I could click on the maximum heart rate coded in red and it would bring me to that day’s max heart data, as I would like to see the activity level of that day*.*” Instead of going through each day for the different data types presented, clinicians felt that this navigation method would be much more intuitive. P5 simply put it “If I click on it, that just takes me to that day’s activity level. That makes it more efficient.”

#### Insight 4.3: HR Page (“Excessive Detail in Raw HR Plot”)

A high-level overview of the HR data such as the hourly average plot was preferred as seen in Figure 7A and then a more detailed plot was preferred, that is, the raw HR plot (Figure 7B). This is because clinicians wanted to see an overview of the data first and then see the details. Participant 1 mentioned the following:

Start off with a big picture, click on it and go deeper, click on it go deeper. I probably would not put the raw heart rate plot first. It may be a good idea for a cardiologist to see the raw heart rate graphs upfront but for me I would like to put it back.

Participant 8 shared similar thoughts and suggested improvements on this plot:

I would think about presenting data in a most aggregate sort of easy to interpret way at first. If I were to present this, I would present a graph of 10 days and they if you put a range, like a light bar for where the range is and if it goes out of range may be color that in red. So, when I look, I can see in seconds this person has hit the target for almost the entire week, so like very high level and easy to interpret data.

In contrast, Participant 1 felt that the raw heart provided a good overview. This is because of the following:

I like the raw heart rate better because for me I do a lot with EEGs and sleep. It just looks more familiar. You can see general trends in a more holistic way and the hourly plot has less info to work with.

#### Insight 4.4: Activity Levels Page (“Activity Levels Are Informative”)

Quantifying activity level information was very informative to clinicians. Participant 6 gave us key details into why he thought that. Figure 7C shows the activity levels page:

I think this will be really helpful and I will tell you why. Before I do any kind of Psychotherapy. I am a big fan of basic selfcare, basic behavior management. I talk about sleep, diet, exercise, and social support with patients. If someone had anxiety or depression, my spiel is if you can get your HR up at least 20 mins a day just enough to break a sweat and get your blood flowing. There is research that shows that it is as effective as any kind of antidepressant in helping with depression. This is great for monitoring that, what I do now is just ask whether you got any exercise or not, did you go for a walk, did you do anything that got your blood pumping a little bit. If you are wearing the watch, I can pull this up, look at it and talk about it. So, I think this is great in that regard.

Participant 6

Even though the information was useful, the pie chart presenting the percentage of activity level done was difficult to interpret because it did not display the time of day the activity was done. Participant 6 shared key insights into improvements in this graph. Participant 8 suggested, “number of minutes would be more helpful because I am thinking 10% of what? Does that mean day, and does that day include sleep time?” In addition, Participant 6 also asked, “Is there a way to know they have taken the watch off?” In addition, Participant 7 suggested the following:

Ways that this might be more helpful would be if this was displayed as a trend over time. So, it would be easy to capture all that data for a week. That gives a high-level overview.

### Workshop 5: High-Fidelity Interactive Prototype

#### Insight 5.1: HR Page (“Identifying Peaks and Trends”)

**Figure 9:**
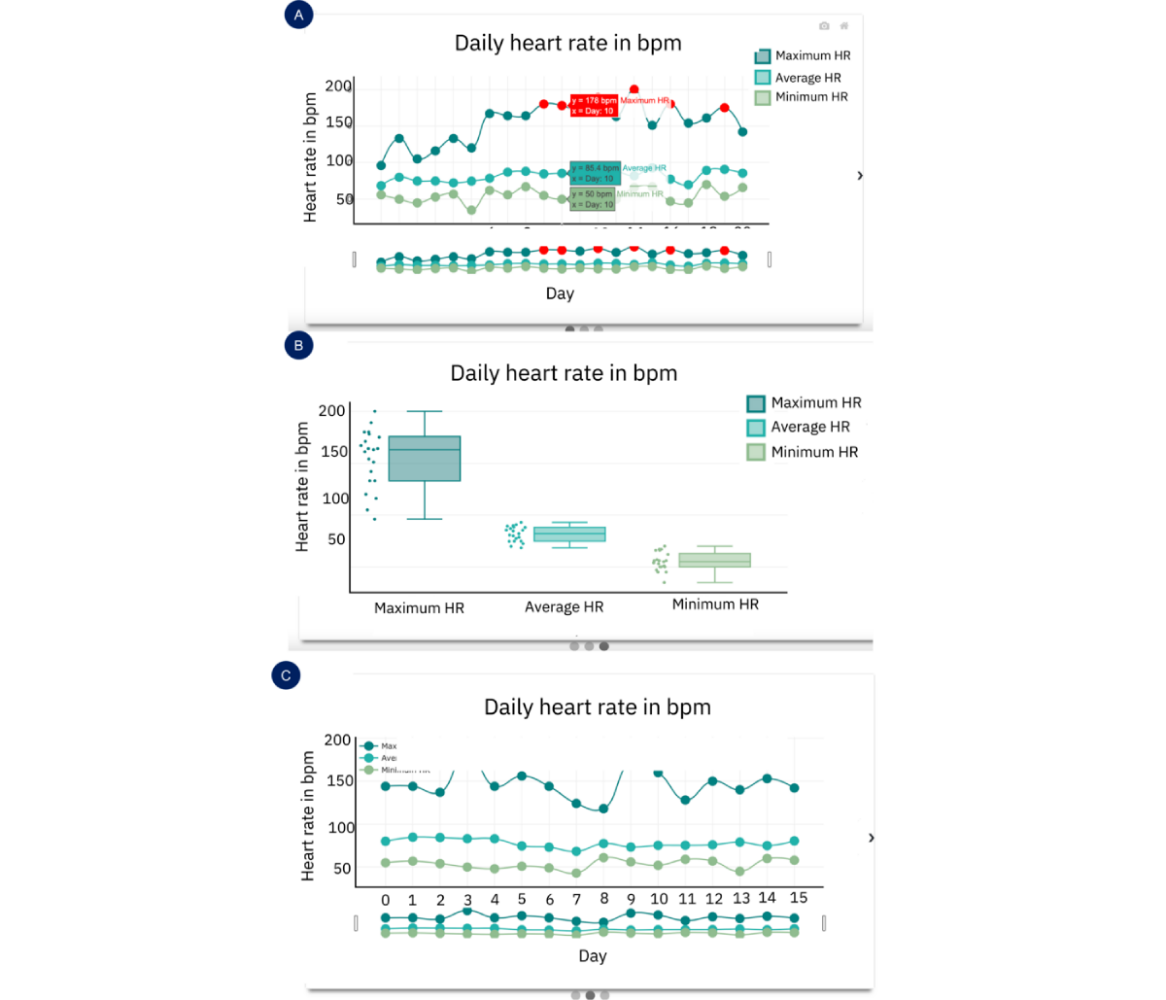
Presented in the workshop: (A) a colored scatter plot for heart rate, (B) a box plot for daily heart rate, and (C) a scatter plot with no color indicators. bpm: beats per minute.

All clinicians preferred to see the first plot, that is, the scatter plot combined with the line plot shown in Figure 9A. Participant 7 and Participant 8 provided some key details regarding why this was the case. Participant 7 said, “It’s easy to look at upfront. I like the fact that some things are red so it drew my eye to those things, so clearly there was something about those days where they were out of range or hit some target*.*” whereas Participant 8 mentioned, “My preference would be to see this one, maybe because I am more used to seeing data this way. I like this because it just highlights certain important ones*.*” Participant 1 also mentioned a similar opinion and said, “I like the data points better, and to me I can see this better. I like this one the best, as it is easy to see what is happening, and I can see across days. I like the idea of being able to hover over them and see the information*.*” In the line plot in Figure 9C, overall trends were visible to clinicians but identifying the peaks was difficult, as Participant 8 said, “I have to go searching for red dots in here*.*” Participant 1 also mentioned, “I like the data points better. I mean I can see the trend here but the peaks don’t stand out.” For the box plot, Figure 9B clinicians were not able to see a trend over the days which is why they did not prefer this plot as Participant 1 puts it, “I guess it’s interesting. The issue I have with it is that it’s harder to get a sense of the days*.*” Participant 8 felt that box plots were more appropriate for research rather than clinical interpretation and said, “I don’t know how I would interpret this clinically. From a research perspective I know what it means.”

#### Insight 5.2: HR Page (“Range Slider Is Useful but Not Intuitive”)

Clinicians in this study appreciated the range slider feature although it was not intuitive at first to them. Participant 1 explicitly said, “I don’t think I would figure that out, what those things are and that I can move those. I thought they were just like a design there, but I like this feature, it’s nice*.*” Participant 8 shared similar thoughts as Participant 1 and said, “I think the ability to hone in on certain timeframes is good, but I think this mechanism is not intuitive. I wouldn’t have been able to figure out that this is until you told me.” Participant 4 and Participant 6 both argued that this feature was very useful but not intuitive. They shared key insights on how to improve this feature Participant 4 suggested, “Probably make that like an arrow or something, make that so it’s obvious that that’s what you are doing*.*” as shown in Figure 9A. Participant 4 also suggested the following:

I think you would have to do some kind of tutorial where you can show the mouse when it hovers over this happens, when the mouse hovers over the reset axis button of the plot this happens, or when you hover over the range slider feature this happens.

#### Insight 5.3: HR Variability Page (Bar vs Line vs Scatter Plot)

Most clinicians preferred to see the bar plot of all the 3 presented plots. The reasons included the following: (1) the data point legend was visible on first sight without having to hover over it and (2) the trend was clearly visible in the bar plot over the scatter plot. In the line plot, even though the trend was visible, clinicians did not want to hover over the line plot to see the data point. All 3 HRV plots are shown in Figures 7D and 7F. Participant 8 shared, “This one [bar plot] I like better because I can see the numbers at a glance.” Participant 1 wanted to see consistency in the presented plots and said, “I don’t have a strong preference for this. I think it might make sense to keep it consistent with the heart rate plot [scatter + line] if that’s what you are going to use.”

#### Insight 5.4: HR Variability Page (“Interpreting HRV”)

**Figure 10:**
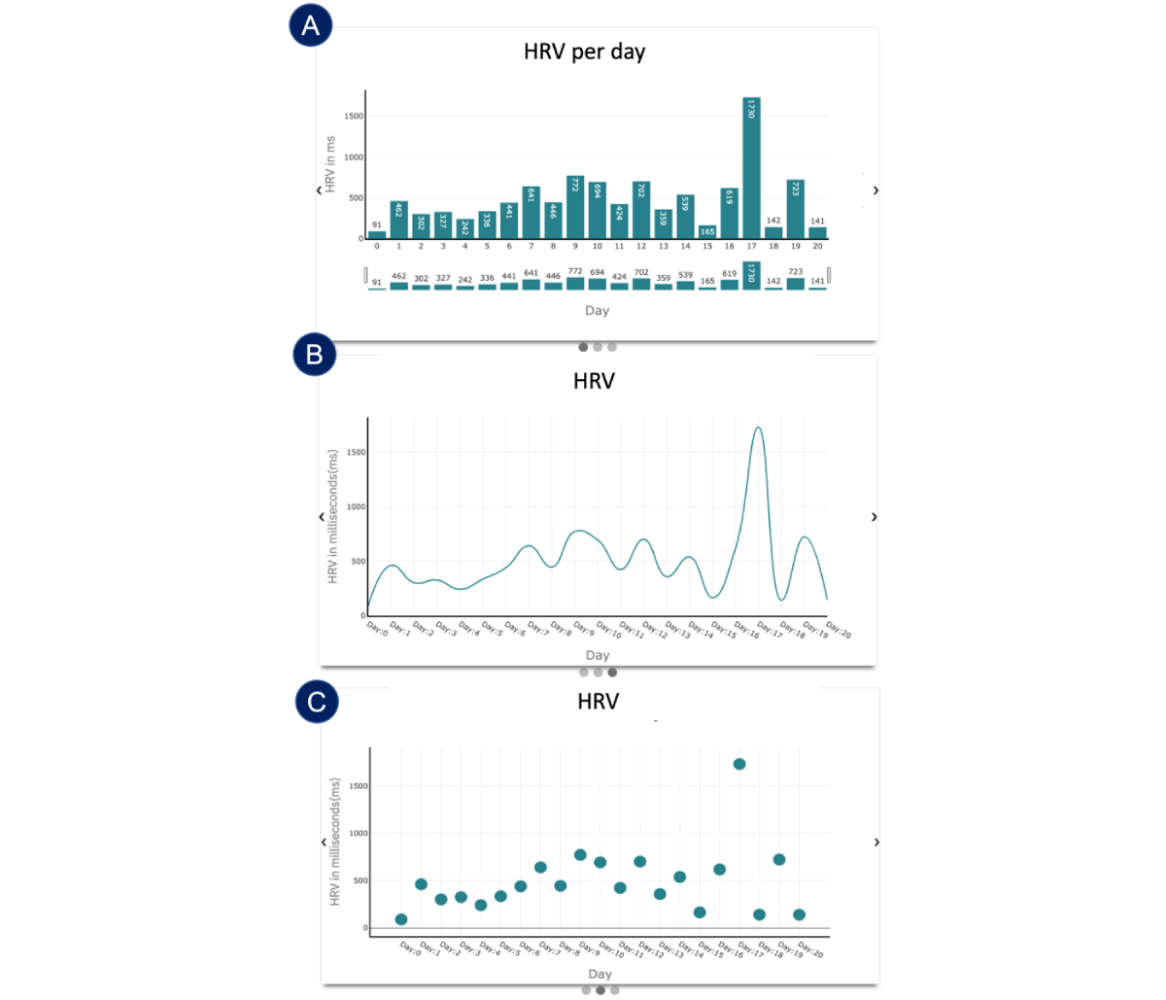
User Experience Questionnaire (UEQ) responses.

One of the concerns during the presentation of HRV as a metric was that clinicians did not know how to incorporate it in daily clinical practice. Participant 8 shared the concern that “HR is a parameter that all physicians are familiar with. HRV is a measure physician don’t really consider in practice. I use it in my research, but you don’t really use it in practice much.” Clinicians also wanted a color-coded plot as well in the HRV bar plot but they debated that it might not make sense as Participant 1 said, “For Heart Rate Variability I know there is a lot more controversy on how to interpret that, what is good, what is bad, so it’s probably harder to code, and may be it doesn’t make sense to do that from the perspective of HRV. I am not an expert on that so I can’t really speak to it.” In the HRV view, clinicians preferred the bar plot over the other plots. Figure 10 shows all three HR variability plots.

#### Insight 5.5: Activity Levels Page (“All in One Plot”)

**Figure 11:**
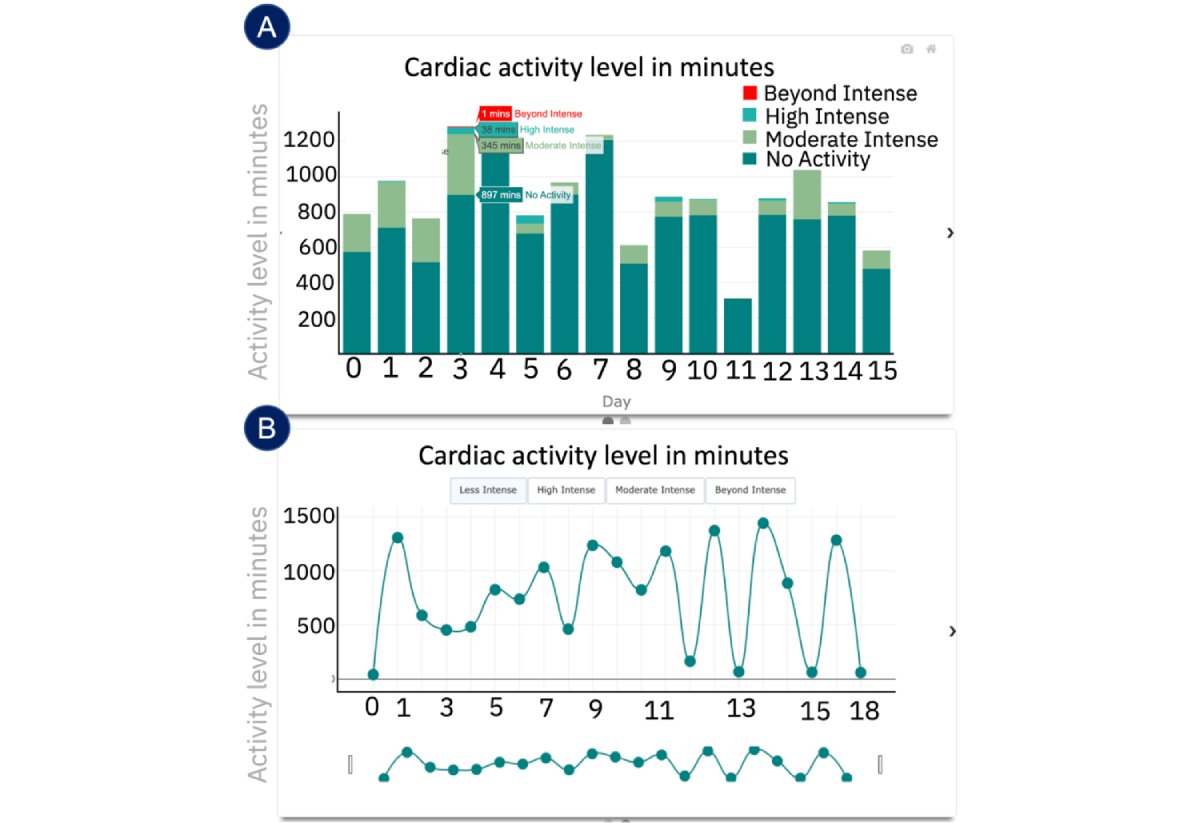
Activity level plots: (A) heart rate (HR)–based activity level in minutes using bar plots and (B) HR-based activity levels using scatter plots.

The consensus of clinicians was that Figure 11A was the preferred plot on the activity levels page. This was because it showed all the data simultaneously. For example, all the 4 levels of activities are visible in the stacked bar plot in Figure 11B, and clinicians did not want to click on the button every time. Participant 1 expressed simply, “I like this chart [Figure 11A] over the other one for this purpose, because everything is all together.” Other clinicians shared the same sentiment, Participant 8 mentioned, “I definitely like this one better. You can just click on something, and you can look at what their whole day looks like.”

### User Experience Questionnaire

**Figure 12:**
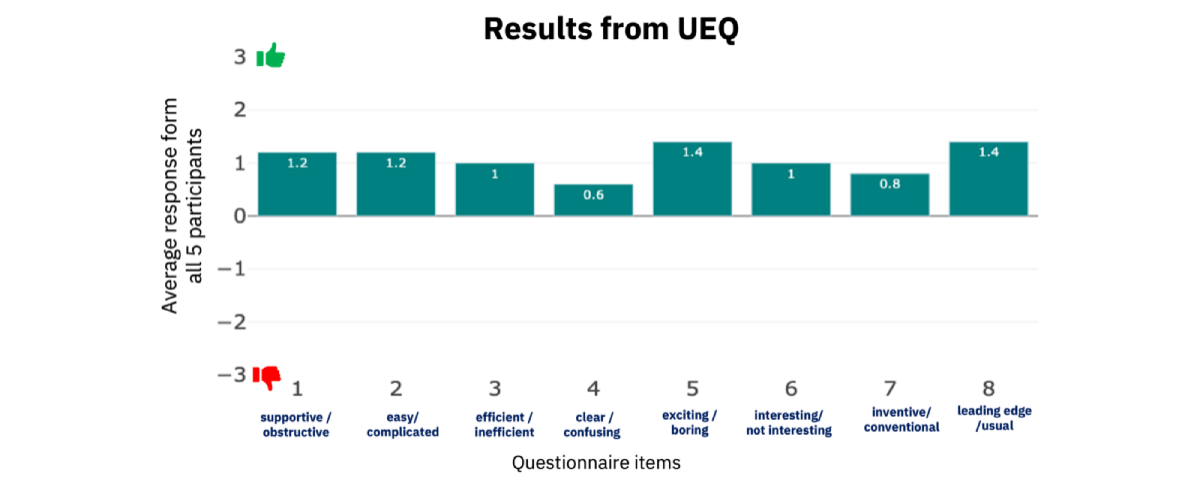
User Experience Questionnaire (UEQ) responses.

The UEQ was conducted by 5 clinician participants (participants 1, 4, 6, 7, and 8). In this section, we analyze the outcomes of the UEQ for each question [21]. Observing the results in Figure 12, we learn that on average, clinicians reported that the CarePortal dashboard was supportive, scoring the application 1.4 on a 7-point Likert-type scale ranging from −3 to +3. A similar positive score was achieved regarding ease of use for question 2: complicated or easy. The highest scores were observed for questions 5 and 9. Clinicians indicated that the application was exciting and toward the leading edge. The lowest score was observed for question 4 (clear or confusing), in contrast with question 2, in which the participants answered whether the application was complicated or easy. We hypothesized that clinicians may have found it confusing to navigate multiple graphs for the same information. This was later removed from the future version of the CarePortal dashboard. Table 3 lists the questions selected from the UEQ [21].

The UEQ benchmark data set contains data from 21,175 people from 468 studies concerning different products (business software, web pages, web shops, and social networks). These data were collected using the full UEQ questionnaire. The benchmark data set is used to compare the hedonic, that is, the pleasing appearance aspect of CarePortal, and pragmatic quality, that is, the practical functionality of the dashboard. Comparing the results with the benchmark UEQ data set we can observe that the pragmatic quality of our application was below average, implying that 50% of the benchmark data set results were better. The hedonic quality was above average, implying that 25% of the benchmark data set results were better. Overall, our app was above average, implying that 25% of the benchmark data set results were better.

**Table 3:** Survey items from the User Experience Questionnaire (−3 and +3 indicate the negative and positive ends of the score that could be rated by the users; n=5).

	Score
Complicated	−3
Inefficient	−3
Confusing	−3
Boring	−3
Not interesting	−3
Conventional	−3
Obstructive	−3
Usual	−3
Easy	+3
Efficient	+3
Clear	+3
Exciting	+3
Interesting	+3
Inventive	+3
Supportive	+3
Leading edge	+3

## Discussion

### Overview

In this study, we designed and implemented an interpretable wearable sensor data visualization dashboard for clinicians with a background in psychology and neuropsychology. We aimed to answer the following research question through this study: “What are the vital elements of a data analytics dashboard that can help to visualize, analyze, and interpret patient wearable data for clinicians to support an informed decision-making process in an easy interpretable way?” To answer this question, we used a participatory design process to identify the needs of clinicians and to involve them in the dashboard design process. We not only analyzed qualitative data from workshops but also implemented CarePortal as a Python Flask data dashboard. Clinicians used the CarePortal dashboard in a remote setting and evaluated its usability.

### Principal Findings

From this study, we concluded that data visualization techniques such as raw HR data (Figure 2) might not be interpretable versus providing an overview of HR data during the day, for example, in Figure 9A. We also learned that clinicians prefer data plots that show trends rather than distribution statistics of the data, as shown in the box plots in Figure 9B. In addition, visualizations that offer an overview of several days of data in a single presentation can be easily interpreted by clinicians. Finally, we conclude that clinicians prefer plots as shown in Figures 9A, 10A, and 12A, and , as these include all the factors discussed earlier. Figure 8 shows the navigational version of the finalized CarePortal dashboard. The final version takes a user from the loading page to the patient page, where a particular patient can be selected. Once the patient is selected, it allows the user to visualize the clinical data. The menu bar on the side allows toggling between data types. In this study, we found that clinicians not only want to see the wearable sensor data but would also want to get context behind what patients are doing, for example, when the HR is high, clinicians want to know whether they are running up a staircase or late for office work or if it is because of a stressful event. Contextualizing wearable sensing data with this type of information can provide clinicians with detailed insights into what is happening when patients do not visit their office.

We found 2 other similar studies that designed wearable sensor data dashboards for (1) neurologists treating Parkinson disease and (2) a gait analysis tool for clinicians. Elm et al [3] found that in a data dashboard for clinicians’ medication compliance, symptoms were the most beneficial feature, followed by the severity of electronic patient-reported outcomes. Activity level and nighttime activity, both sensor-derived data points, were the least beneficial components for clinicians but still supported the clinical assessment two-thirds of the time. Similar to our dashboard, Elm et al [3] presented hourly and daily metrics, these daily metrics included trends over a period of several days, whereas the hourly metrics included symptoms present over an hour. Seals et al [12] developed a clinician-centered dashboard for gait analysis, in which they found that clinicians prefer to have a longitudinal view. They wanted to understand the trends in how patients performed the walking test during different seasons such as winter and summer. Although this work provides insights into clinicians’ data visualization preferences, we observed a common theme during this participatory design study with the previously described studies: clinicians not only want to see quantitative data but also would like to see an overlay of qualitative self-reports by patients. This is because a high HR can be one of the factors indicating stress level; however, it is not the only one. For example, Participant 2 mentioned, “The high heart rate can be because of the person or running or walking up the stairs.” Therefore, it is important to know what the person is doing when the HR is high. A tool that displays this type of information can be a powerful source of information. In the future, we would like to incorporate accelerometer-based activity data along with HR data to offer additional context to clinicians for improved decision-making. Nevertheless, the CarePortal seemed promising to clinicians, providing insights related to patients’ daily health and activity markers to improve clinical decision-making.

### Limitations

Although we obtained promising results for interpretable visualization techniques, there are some limitations to our study. In this work, we only present wearable sensor data to clinicians, as [3] suggested that electronic patient-reported outcomes along with medication data are most beneficial; in this work, we did not introduce this in the final version of the dashboard due to the lack of data availability. Another limitation of this study is that we do not yet integrate CarePortal into the EHR system from which we can integrate other health data of patients, such as blood pressure. During our first workshop, we found that clinicians used separate EHR platforms during their practice, and they were not integrated with each other; therefore, it was not possible to obtain data directly from their EHR system. In this work, we only presented HR data from a smartwatch; however, in today’s world, wearables can provide much more information, such as blood pressure, sleep quality, and sedentary information, and integrating these data can prove to be valuable for clinicians. Another limitation of this study was that we did aim to explore how confident clinicians would be with the wearable data presented to them. Therefore, we aim to explore how many such inputs clinicians can deal with in a real-world setting in further studies and determine the confidence level of clinicians in viewing such wearable data in their day-to-day practice.

## Abbreviations

EHR: electronic health record
HIPAA: Health Insurance Portability and Accountability Act
HR: heart rate
HRV: heart rate variability
IRB: institutional review board
UEQ: User Experience Questionnaire
UI: user interface
UX: user experience

## Data Availability

As the study participants who helped collect the wearable data did not consent to share the data beyond the research team, the authors are unable to share the wearable sensor data set at this point.
